# Sustainability in Supply Chain Management: Aggregate Planning from Sustainability Perspective

**DOI:** 10.1371/journal.pone.0147502

**Published:** 2016-01-25

**Authors:** Metin Türkay, Öztürk Saraçoğlu, Mehmet Can Arslan

**Affiliations:** Department of Industrial Engineering, Koç University, İstanbul, Turkey; Middlesex University London, UNITED KINGDOM

## Abstract

Supply chain management that considers the flow of raw materials, products and information has become a focal issue in modern manufacturing and service systems. Supply chain management requires effective use of assets and information that has far reaching implications beyond satisfaction of customer demand, flow of goods, services or capital. Aggregate planning, a fundamental decision model in supply chain management, refers to the determination of production, inventory, capacity and labor usage levels in the medium term. Traditionally standard mathematical programming formulation is used to devise the aggregate plan so as to minimize the total cost of operations. However, this formulation is purely an economic model that does not include sustainability considerations. In this study, we revise the standard aggregate planning formulation to account for additional environmental and social criteria to incorporate triple bottom line consideration of sustainability. We show how these additional criteria can be appended to traditional cost accounting in order to address sustainability in aggregate planning. We analyze the revised models and interpret the results on a case study from real life that would be insightful for decision makers.

## Introduction

Supply chain management has become one of the most important core functions of companies in manufacturing and service sectors. Supply chain management considers all of the stages from raw material procurement to consumption by the end users in fulfilling the demand for a certain product or service. In the simplest terms, whenever there is a demand either for a product or a service and supply to fulfill this demand, a supply chain emerges.

“The supply chain encompasses all activities associated with the flow and transformation of goods from raw materials stage (extraction), through to the end user, as well as the associated information flows. Material and information flow both up and down the supply chain.”[1]

In particular, a supply process receives certain inputs that are then turned into desired outputs via a transformational process to satisfy the demand. Therefore, any activity in fulfilling the demand is in the scope of the supply chain system under consideration. Any business entity either being a manufacturing or a service business has an underlying supply chain system and in the core of this system is what is called the production or operations function of the business. Production and operations functions focus on the flow of information, and material between stages of the supply chain.

Complexity of supply chains shows significant varieties depending on its structure, products and services provided by companies. For example, Apple Corp. is considered to manage one of the most efficient supply chains in the world these days. The supply chain of Apple starts with the R&D phase where new products and services are designed and the design concepts are tested. Then, in the Pre-Launch stage involves mainly building up inventories for the products in accordance with the expected demand. The launch stage involves forecasting the demand for the next two quarters and resolving backlog problems. The quarterly reviews focuses on managing inventory levels, adjusting demand forecast and monitoring sales, demand and product life cycle. The last three stages that include Pre-Launch, Launch and quarterly review is considered as part of the Aggregate Planning activity. The supply chain management problems deal with long-term, medium-term and short-term decision. The medium-term decisions affect the system performance between 3–9 months and generally include the capacity utilization, workforce management and inventory management decisions. In general, these decisions are considered simultaneously in aggregate planning problem [2]. The aggregate planning problem sets production capacities, labor utilization, inventory profiles, backordering in the case of not being able to satisfy the demand on time, and subcontracting. Therefore, the aggregate planning problem is a key instrument in applying the strategic level decisions while determining the principles of operational level problems. In addition, the aggregate planning problem encompasses economic, environmental and social factors.

In managing the supply chains, integrating sustainability in the decision-making process had become one of the focal subjects due to increasing awareness from the customers who use the products supplied by companies.

In this paper, we propose a methodological approach to incorporate sustainability considerations in the aggregate planning problem of supply chain management and illustrate our approach on household refrigerator industry in Turkey.

### Sustainability

Sustainability is used either as the ability to sustain a practice or process or to refer to environmental consciousness in the literature and most non-academic resources. Both comprehensions are valid but incomplete. Sustainability is closely related to the concept of sustainable development, which is defined as “the development that meets the needs of the present without comprising the ability of future generations to meet their own needs” [3].

The traditional method for posing the aggregate planning problem is based on a purely economic model. However, sustainability suggests that environmental and social criteria need to be considered along with economic criterions, which are called the “three pillars” or the “triple bottom line” of sustainability [4]. To address the sustainability in supply chain management, the decision maker should incorporate these three pillars of sustainability simultaneously into the decision-making process. The application of the triple bottom line accounting on economic order quantity (EOQ) was shown to provide very useful insights [5]. It was shown that inclusion of environmental and social factors in addition to economic considerations allows the decision makers to assess their decisions from sustainability perspective.

In this paper, a methodological approach to incorporate sustainability considerations in the aggregate planning problem is presented. The standard model is extended to according to additional environmental and social criteria. The revised models are then analyzed to drive insights into the aggregate planning problem from sustainability perspective. The analysis shows how environmental and social criteria can be appended to traditional cost accounting in order to address sustainability in supply chain management with the aggregate planning problem.

The literature on sustainability of supply chains focus on reviews and also a number ideas to incorporate sustainability. However, most of the existing literature considers only economic and environmental aspects of sustainability. Beamon [6] and Handfield et al. [7] both present a review of environmental management issues in supply chain planning and provide a framework of achieving and maintaining a greener supply chain. These papers consider sustainability from environmental perspective only. They analyze the impact of environmental policies on the supply chains. They show that environmental policies must be considered in the decision-making framework in order to have greener supply chains. Toptal et al. [8] analyze different regulations for carbon emission policies. An important consideration is the cap-and-trade policy that allows trading of carbon emissions through a system such as EU Emissions Trading System. The firms have two options: they either buy or sell carbon allowances at a specified market price or they pay their carbon emissions costs as taxes. Boulanger and Breechet [9] survey the genetic methods that can be used by the policy-makers in assessing the sustainability performance of their policies. They also discuss the appropriateness of these methods using sector-based applications and conclude that the multi-agent simulation models are best suited with decision-making for sustainability. Turkay [10] reviews methods for environmentally conscious supply chain management and categorizes them as product centric (closed-loop supply chains), production system centric (environmentally conscious production) and transportation system centric (sustainable transportation) approaches. Under environmentally conscious production, supply chain systems with environmental extensions are studied. Carter [11] deals with purchasing social responsibility (PSR) as an application of corporate social responsibility (CSR) to supply chains. Carter and Jennings [12] analyze the effect of social responsibility projects on supply chain performance and conclude that these projects enhance the supply chain performance. The main message in [11] and [12] is the necessity to include social factors in the models in order to achieve sustainability along with the environmental factors. It is clear from the literature that inclusion of social factors in the aggregate planning of sustainable supply chains has not attracted attention and we aim to fill this gap in this paper.

### The Aggregate Planning Problem

The standard aggregate planning problem aims to determine the production levels, inventory kept in the supply chain, hiring and firing employees, overtime production, backorders and demand satisfaction levels with the objective of having the minimum cost or maximum profit. It is the most widely solved supply chain management problem. In this paper, the standard aggregate planning model is extended to include carbon footprint, energy consumption, employee job-security and morale-motivation, employee health and work-family balance, and customer satisfaction considerations with the purpose of incorporating the triple bottom line accounting of sustainability. The carbon footprint consideration is quantified by GHG emissions and the energy consumption consideration by the electricity/heat usage metrics during manufacturing. On the other hand, hiring and firing quantify employee job-security and morale-motivation factors whereas employee health and work-family balance factors are considered by the overtime hours. Finally, the customer satisfaction consideration is quantified by the fill rate of the demand metric. Based on these metrics we develop six models: two environmentally revised models (emissions cap and energy consumption cap), three socially revised models (smoothing and layoff limits, overtime limit, and service level target), and one TBL accounting model. For all of the revised models, mathematical programming formulations are analyzed with data from real-life along with numerical sensitivity analyses.

In aggregate planning, the decision maker first estimates aggregate cost components including labor costs, capacity changing costs, production costs, inventory holding costs, stock-out and backlogging costs, and subcontracting costs. The estimation of these costs is not an easy task; however, it is a prerequisite for aggregate planning. Apart from the cost components, other factors including the demand forecast, *D*_*t*_, for each period, *t*; the number of working days in each period, labor hours required per item of production, and the initial inventory, backlog and workforce levels are required to devise the aggregate plan. Once all these inputs are determined, the aggregate planning problem can be formulated as a mathematical programming problem and solved. The resulting plan should then be disaggregated to form Master Production Schedule (MPS) and obtain the Materials Requirement Plan (MRP) [13]. In other words, the aggregate planning is a prerequisite for many operations including production planning, scheduling, and inventory management.

The decision maker not only reveals information on production, inventory, and capacity levels but also on the required capital, machinery, and warehouse space, sourcing decisions and supplier purchase level, customer service levels, and product pricing by deducing the aggregate plan. Clift [14] proposes metrics for sustainability in all aspects; i.e. economic, environmental and social. The author argues that social metrics are not so common and a public consensus is needed to define and use them in decision-making processes. Moreover, aggregation across the dimensions such as expressing ecological impact through monetary units is both unnecessary and undesirable. Koplin et al. [15] provides a case study of an automobile company regarding environmental and social impacts management and present a sustainable supply chain management concept. Sarkis [16, 17] presents a decision-making framework for environmentally conscious business management using analytic network process (ANP). Arslan and Turkay [5] examine the sustainability considerations in the inventory management problems. They include the economic, environmental and social factors of the triple bottom line accounting of sustainability in the deterministic inventory management problem using the EOQ model. Bonney and Jaber [18] provide a list of non-cost metrics for incorporating environmental footprint in the inventory context. Although [18] uses similar models that are available in [5], [18] focuses on the environmental factors of the EOQ model. Cai et al. [19] present a dynamic linear programming (LP) model for large-scale energy generation plants in Waterloo region of Canada. They observe the trade-offs between system costs and GHG emission for the system under consideration. They also state that their model enables analysis of alternative technologies over a number of periods and may be extended to cover a large variety of complexities common in energy systems. Letmathe and Balakrishnan [20] present a linear and a mixed-integer program for firms to determine the optimal product mix and production quantities under environmental constraints in addition to the production constraints. Lineras and Romeo [21] give a multi criteria decision-making method for electricity planning and model GHG emissions as well as radioactive waste as minimization objectives. Penkuhn et al. [22] present a constrained nonlinear program (NLP) for a production planning problem in the process industry.

It is necessary to consider the social factors in addition to the environmental and economic ones in order to analyze supply chains from sustainability perspective. Past literature on sustainable supply chains mainly focuses on the environmental factors. The main conclusion from these papers is to include carbon tax as the environmental factor that gives favorable results in terms of environmental dimension of sustainability. The social factors, on the other hand, has not attracted attention due to lack of understanding and data on their impact on the performance of sustainable supply chains.

This paper presents three novel contributions to the aggregate planning problem: the introduction of sustainability through triple bottom line accounting that includes social aspects in addition to economic and environmental aspects, the use of the learning curve in reflecting the workers performance on the production efficiency with time, and the smoothing limit that decreases the hiring and firing employees on the system performance. Then, the effects of all of the aggregate planning decisions with the realistic model are analyzed from sustainability perspective. From environmental perspective, carbon footprint and carbon tax are added to the classical model. Carbon footprint is used for greenhouse gases emission per unit production and its cost effect is calculated by carbon tax perspective. The electricity usage is also added to traditional model from environmental perspective because electricity used for production is a major source of environmental pollution. This situation is also highlighted by the fact that electricity generation processes in developing counties like Turkey, India and China are focusing on coal, oil and gas power plants. Layoff limit is considered in the model to incorporate the social perspective of sustainability and to analyze the effect of higher number of worker hiring and firing due to negative effect on coordination of the workers.

In this paper, the learning curve is considered as an integral part of the aggregate planning process to reflect the performance improvement of newly hired workers realistically. Learning curve implies that workers who are newly hired cannot achieve high performance before becoming proficient in their tasks. The experts of worker performance assessment and monitoring agree that a newly hired worker can achieve the maximum performance level after they work for approximately one year. Adler and Clark [23] state that experience of the workers affect the total production capacity. According to their findings, the production quantity of the previous month determines that of the next month. A newly hired employee works at 70% of maximum performance for first three months. Between the third and sixth months, hired workers perform at 80% on the average. For the rest of the 6 months before complete first year, they work at 90% performance level. Learning curve helps to model the problem more realistically instead of using hired workers at maximum performance level that contradicts with the reality. In addition, layoff of experienced workers and substituting them with inexperienced workers leads to lower production capacity utilization in the manufacturing systems. Learning curve is one of the original contributions of this paper to the aggregate planning problems.

Smoothing limit is another novel concept that we add to the aggregate planning model to reflect reality. Smoothing limit decreases the effects of hiring or firing more people based on the initial number of workers. If a large fraction of the employees are laid off, this causes a significant decrease in the morale and motivation of workers. When a large number of new employees is hired, the coordination among the workers decrease and this can cause conflict between old and new workers leading to a decrease in the performance of workers. We consider a smoothing limit of 35%: the total number of fired and hired people during a year cannot exceed 35% of initial number of workers. There is also smoothing limit for each month as 10%.

The aggregate planning is a fundamental step in the entire supply chain and operations management. In this paper, the methodological approach to incorporate sustainability considerations in the aggregate planning problem is presented. The standard model is enhanced with realistic considerations including the learning curve of new employees and smoothing limits on the hiring and firing of employees. Moreover, the aggregate planning model is revised to incorporate environmental and social effects to conduct a triple bottom line accounting analysis of sustainability and the solutions obtained with the model are analyzed to obtain insights. The analysis shows how environmental and social criteria can be appended to traditional cost accounting in order to address sustainability in supply chain and operations management based on the aggregate planning problem. The sensitivity analysis is done to understand the effects of carbon cap, overtime limit, smoothing limit, and service level on total cost. And each of these analyses is done according to different subcontracting limit (between 0 percent and 100 percent) on the demand for each month.

## Materials and Methods

In this section, we discuss the aggregate planning problem in detail, provide discussion of the sustainability metrics that we use, and also the case study that we use to illustrate the concepts and results.

### Aggregate Planning Problem

The standard aggregate planning model considers all of the available cost components related to aggregate plan. The model sets the production capacity according to regular and overtime production. Hiring or firing employees to meet the customer demand with the minimum total cost is usually practiced to balance the workforce according to demand for the products and services. Inventory level is another aspect of the aggregate plan: inventory level is a function of production, subcontracting and stock-out. The cost function defined by these cost components that needs to be minimized at optimality.

The standard mathematical programming formulation of the aggregate planning problem is given below (please refer to S1 Table for the notation used in the model):

$$\text{min}z=\sum_{i=1}^{T}hn_{t}c_{L}W_{t}+c_{m}P_{t}+c_{o}O_{t}+c_{s}S_{t}+c_{su}SU_{t}$$ (1)

s.t.

$$P_{t}\leq Kn_{t}W_{t}+O_{t}\;\forall t\in \left\{1,\ldots .,T\right\}$$ (2)

$$W_{t}+F_{t}=W_{t-1}+H_{t}\;\forall t\in \left\{1,\ldots .,T\right\}$$ (3)

$$I_{t}+D_{t}=P_{t}+S_{t}+SU_{t}+I_{t-1}\;\forall t\in \left\{1,\ldots .,T\right\}$$ (4)

$$O_{t}\leq Kn_{t}W_{t}\;\forall t\in \left\{1,\ldots .,T\right\}$$ (5)

$$W_{t},H_{t},F_{t}\in \left\{Z^{+}\cup \left\{0\right\}\right\}\;\forall t\in \left\{1,\ldots .,T\right\}$$ (6)

$$P_{t},O_{t},I_{t},SU_{t},S_{t}\geq 0\;\forall t\in \left\{1,\ldots .,T\right\}$$ (7)

$$W_{0}=W_{init}$$ (8)

$$I_{0}=I_{init}$$ (9)

We use this standard model as the base case and compare the change in the optimal solution when the sustainability considerations in terms of environmental factors and social factors are considered. Since this model is a multi-period problem, production amounts, inventory levels, the number of workers and most importantly demand for product can change every period. So, decisions for each period must be determined separately. However, the nature of supply chain management dictates that the decisions at each period are affected by the decisions in the previous period and the decision at each period affect the decisions in the subsequent periods.

### Sustainability Considerations

The traditional aggregate planning model only considers the economic aspects of aggregate planning. However, social and environmental aspects had become an important issue about supply chain in the 21^st^ century. Decision makers should integrate environmental and social aspects in addition to economic aspect to accomplish sustainability in supply chain.

#### Economic Factors

The economic factors of aggregate planning are the first step of sustainability approach. These factors consist of raw material costs, inventory holding cost, labor production cost, overtime cost, backlog cost, and subcontracted cost of product. These cost elements constitute the economic side of sustainable aggregate planning problem.

#### Environmental Factors

The environmental factors that affect the aggregate planning decision are examined in this subsection.

**Carbon Footprint:** Carbon footprint refers to the set of greenhouse gases including carbon dioxide released by an organization [24]. In order to assess the environmental performance of an organization, the amount of GHG emissions is commonly used in the green/environmentally friendly supply chain management literature.

The cap on the amount of GHG emissions released by the organization during the entire planning horizon, as in the case of Kyoto Protocol [25], is given by *ε*_*C*_. The parameter, ${c^{\prime }}_{I}$ denote the amount of GHG emissions due to inventory holding, ${c^{\prime }}_{P}$ denote the amount of GHG emissions due to regular time production, and ${c^{\prime }}_{S}$ denote the amount of GHG emissions due to subcontracting. Then, the total GHG emissions from the aggregate planning over the planning horizon *T* is given by,

$$\sum_{i=1}^{T}c_{I}^{\prime }I_{t}+c_{p}^{\prime }P_{t}+c_{su}^{\prime }SU_{t}\leq \varepsilon_{C}$$ (10)

In this paper, we base our calculations for each product on the amount of GHG emission for 1 million dollar production of products. The unit cost of CO_2_ is known [26] and the cost of GHG emissions per product can be estimated by multiplying the amount of CO_2_ when producing one product. We added this cost as an environmental cost to the problem and we set an upper bound for total CO_2_ amount released to air.

**Energy Consumption:** One of the fundamental considerations in environmental impact assessment is the amount of energy used by the organization. Energy, particularly in the form of electricity, is a fundamental entity that is consumed in most industrial operations [6, 9, 27]. We consider a cap, *ε*_*E*_, on the amount of electricity used by organization.

The parameter ${c^{\prime \prime }}_{I}$ denote the amount of electricity used due to inventory holding, ${c^{\prime \prime }}_{P}$ denote the amount of electricity used due to regular time production, and ${c^{\prime \prime }}_{S}$ denote the amount of electricity used due to subcontracting. Then, the total electricity consumption is given by,

$$\sum_{i=1}^{T}c_{I}^{\prime \prime }I_{t}+c_{p}^{\prime \prime }P_{t}+c_{su}^{\prime \prime }SU_{t}\leq \varepsilon_{E}$$ (11)

#### Social Aspects

The social factors that affect the aggregate planning decision are examined in this subsection.

**Employee Job Security and Morale-Motivation:** One of the standard practices in aggregate planning is hiring and firing of employees at some periods depending on the demand for products, and the available inventory. This in return requires regular changes in capacity, which eliminates job security of the employees, impairs their morale-motivation and leads to voluntary retention [7]. To avoid decrease in the morale-motivation of employees, an upper limit on the total number of fired and hired employees during the entire planning horizon must be considered. The workforce smoothing limit (*S*_*lim*_) can be defined in Eq (12) and this limit can be changed to observe the sensitivity of the system to this limit. The following constraint is added to the standard aggregate planning model to account for employee job security and morale-motivation:

$$\sum_{i=1}^{T}H_{t}+F_{t}\leq S_{\text{lim}}$$ (12)

Furthermore, an upper bound on the number of employees fired at each period of the planning horizon (*L*_*lim*_) may also be imposed with the following constraint:

$$\sum_{i=1}^{T}F_{t}\leq L_{\text{lim}}\;\forall t\in \left\{1,\ldots ,T\right\}$$ (13)

The information on firing, hiring and overtime costs are defined by company in the case study that we consider in this paper. We included production labor cost as a social cost beside economic cost because it effects the employees and it needs to be considered as social cost since people are employed in the production processes.

**Employee Health and Work Family Balance:** The standard aggregate plan also allows overtime work for employees under the time flexibility strategy. This provides overtime working during peak in demand periods and this helps the company in achieving a high service level for the customers. However, overtime has physical and psychological strain on employee health [28, 29]. Moreover, extended number of hours worked creates additional risks for accidents and damages the social life and work-family balance of the employees [30]. For this reason, labor rights limit the overtime working hours [International Labor Organization (ILO)].

The daily limit of overtime hours for each worker is given by the parameter, *O*_*lim*_; then, the limit on overtime production is given by modifying Eq (5) to:

$$O_{t}\leq \frac{Kn_{t}W_{t}}{h}O_{\text{lim}}\;\forall t\in \left\{1,\ldots .,T\right\}$$ (14)

The overtime limit is given by company as 15% of the working hours for each worker. Manufacturer does not exceed 15% to establish family and work balance.

**Customer Satisfaction:** The standard aggregate planning formulation allows stock-outs when the production is not sufficient to meet the demand. The stock out is available for the traditional model and has a unit cost, *c*_*B*_. However, stock out is not desirable by customers since they will not be able to meet their demand on time. Besides, if the products are related to physical health and safety, stock out is not option. Therefore, the minimum service level is economically desirable not for producer only but also a requirement for the customers. The desired service level as the fraction of the demand satisfied at each period is represented by *α*. Then, the following constraint models the customer satisfaction:

$$S_{t}\leq \left(1-\alpha \right)D_{t}\;\forall t\in \left\{1,\ldots .,T\right\}$$ (15)

Each company has a target service level for its products that helps company to satisfy customer needs at desired level. Higher service level constitutes higher customer loyalty for companies. Therefore, service level of company is one of the key points of production.

### Model Assumptions

In this model, we have the following assumptions:

1. Social factors are added to the model as constraints in the form of smoothing, overtime limit and demand satisfaction. Social cost is defined as a function of worker related costs only in the production system.
2. The purchase cost of electricty includes the carbon tax; so there is no need for adding carbon tax for electricity.
3. There is no back ordering in the supply chain. The lost demand cannot be recovered in the subsequent months.
4. Subcontracting incurs a carbon tax. When a subcontractor charges the total cost, the same carbon tax to each product applies whether it is manufactured or subcontracted.
5. The learning curve is the same to all newly hired workers regardless of the classification of workers.
6. Hiring cost includes training costs for workers and social security payments at the initial step. Firing cost includes indemnity payment for the workers as avarage.

The validity of the above assumptions were tested with two independent manufacturers.

### Case Study: A Refrigerator Manufacturer in Turkey

Refrigerator industry has been steadily increasing its production capacity for the last 15 years in Turkey reaching 7.5 million units produced per year. About 5.5 million of this production is exported to mainly UK, Central Europe and Eastern Europe. We consider a company that has a share of 33% in the domestic market.

Sustainability has become one of the most critical issues for refrigerator manufacturers in Turkey. They have to reduce environmental pollution and increase social welfare for workers to sustain their presence in the market. To achieve sustainability, companies started to take action such as using materials that have lower total GHG emission than previous materials from environmental perspective, understand the problems faced by the workers and increase social welfare of workers. The data for the case study is provided in S2 Table.

### Mixed-Integer Programming

The mathematical programming model presented in the previous section includes both continuous and integer decision variable. Continuous variables can take any value and integer variables are restricted to only integers as long as the constraints given in the model are satisfied. This type of problems is categorized as mixed-integer programming (MIP) problems. Special algorithms must be developed for MIPs, in order to make sure that the integer variables take the proper integer values at the optimal solution. Solving a mathematical programming problem that contains integer variables as a linear programming problem and rounding the integer variables to the nearest integer is not possible due to infeasible and non-optimal solutions. In addition, the aggregate planning problem presented I this paper contains multiple objectives to reflect the triple bottom line accounting of sustainability. The *ϵ*–constraint method is used for solving the multiple objectives models to determine the effect of each objective function on other objective functions [31]. GAMS [32] optimization platform and CPLEX [33] solver can be used to solve the MIP problem. The optimization model and the implementation of the *ϵ*–constraint method in the GAMS optimization platform are given in S1 Model.

## Results

**Fig 1:**
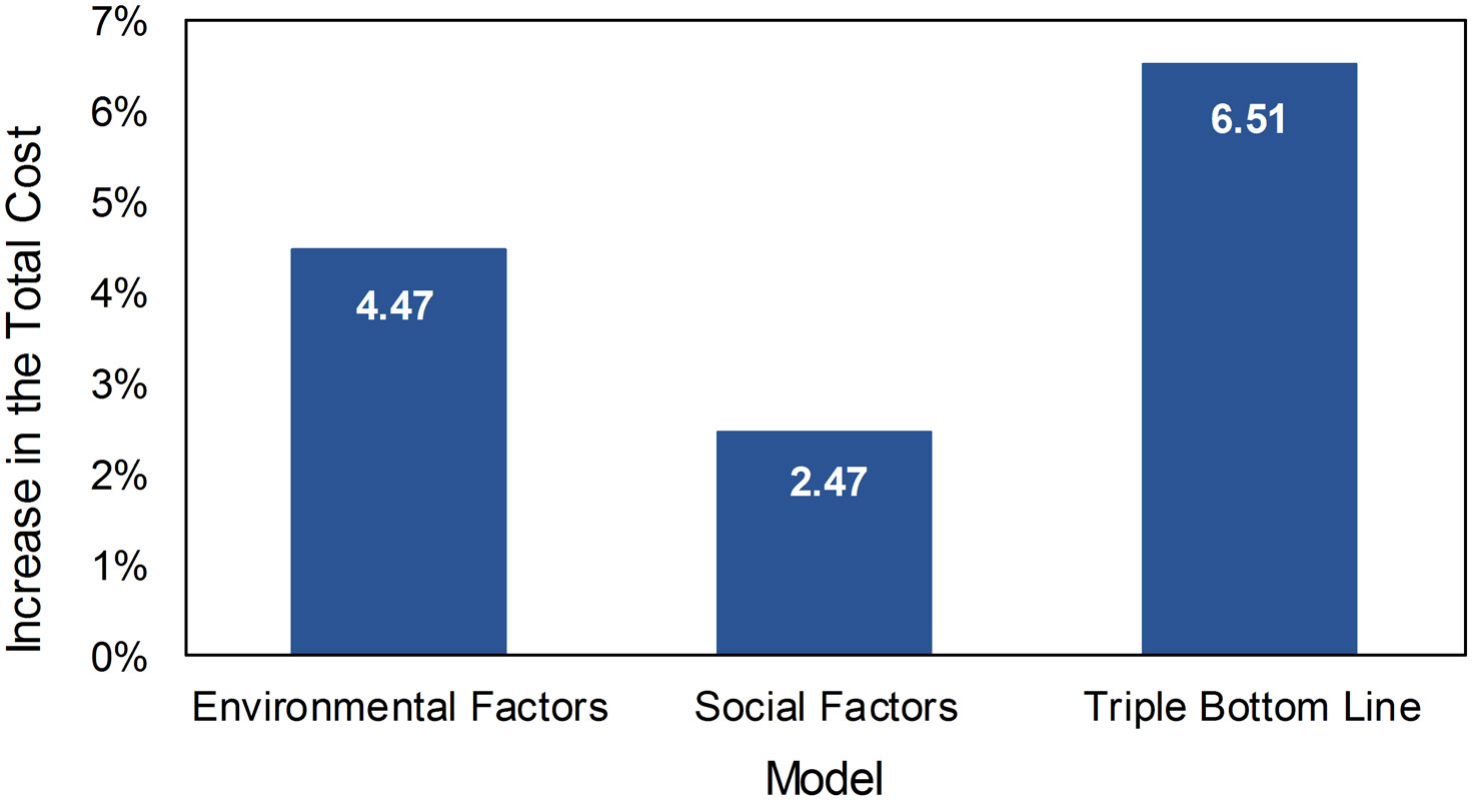
Percentage Change According to Different Models.

We present the results of the sustainability analysis for the case study in this section. First, the effects of social and environmental factors on the total production cost are discussed and a comparative analysis of the results from the Standard Aggregate Planning Model with the results from the models those including sustainability considerations are compared. Next, carbon footprint from production operations and electricity consumption are added to the model in order to analyze the impact of the system on the environment. Then, the model is solved and the results are analyzed by including social factors only to understand the impact of operations on the social side. Last, all social and environmental factors are included to analyze the aggregate planning from the triple bottom line accounting of sustainability perspective. It is important to assess the change in the cost structure when environmental and social factors are considered together with the standard aggregate planning problem. Fig 1 shows the change in the total cost when the environmental and social factors are included separately and the triple bottom line accounting is included for the case study of the refrigerator industry in Turkey.

As shown in Fig 1, when the environmental factors alone are included the increase in the total cost is 4.47% relative to the standard model. On the other hand, the social factors incur an increase of 2.47% in the total cost over the solution of the standard model. These results indicate that the inclusion of environmental factors (carbon footprint and electricity consumption) increase the total cost more than the social factors (worker smoothing limit, layoff limits, and limits on the overtime work hours). For the case study, the demand for products and also carbon emissions per unit production are high, the environmental cost is higher. This result is expected to be similar for energy intensive production sectors. The increase in the total cost when the social factors alone are considered is mainly due to seasonal demand profile (max demand is 291,000 units/month and the minimum demand is 157,000 units/month). In the standard model, it is possible to lay-off workers when the demand is low and also new workers are hired during high demand season without paying attention to workforce smoothing limits. However, the model with social considerations sets a limit on the number of workers hired and fired in each period, the worker turnover cannot exceed 10% of the total workforce and the change in the number of employees in a year could be at most 35%.

When all of the factors of the triple bottom line accounting method (with social and environmental aspects simultaneously) are considered, the increase in the total cost is 6.51%. This change is lower than the total change when environmental and social factors are implemented separately. Therefore, this case study indicates that the implementation of the triple bottom line accounting as a whole increases the total cost by a smaller margin than when the environmental and social factors were implemented separately (6.51% versus 4.47+2.47 = 6.94%). The reason for smaller increases in total cost when triple bottom line accounting is implemented is mainly due to subcontracting. When only environmental factors are included in the standard model, the manufacturer does not use subcontracting and hire and fire workers without any constraint. When the social considerations are included in the model, manufacturer uses subcontracting and this leads to decrease in environmental, social, and production cost while the increase in the subcontracting cost is less than the increase in other costs such as raw material, social, production costs.

We also conduct a sensitivity analysis of the results with respect to important parameters of the model. We identify carbon cap, workforce smoothing limit, overtime limit and service level as important parameters and provide a thorough analysis of the results with respect to changes in their values.

### Carbon Footprint Capacity

**Fig 2:**
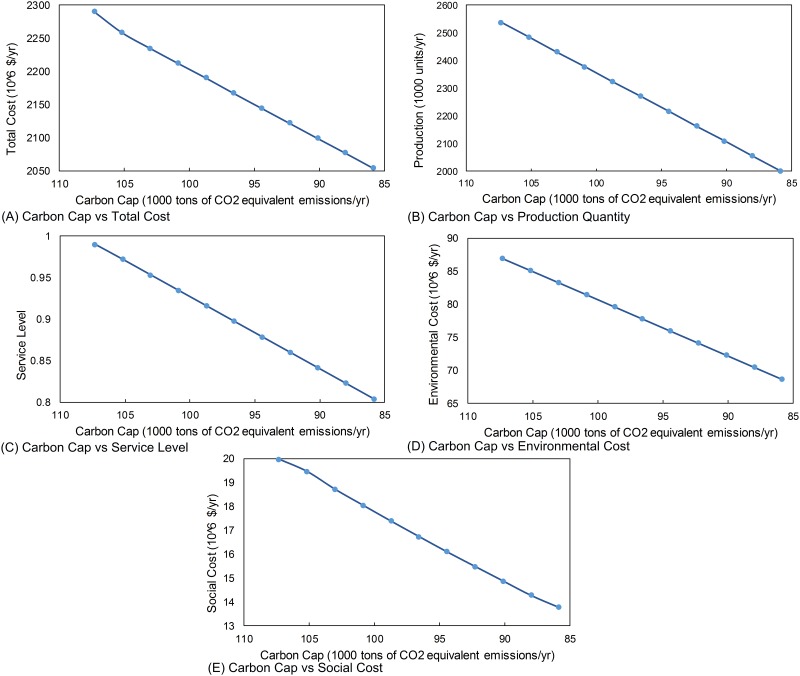
Sensitivity with respect to carbon cap. (A) Carbon Cap vs Total Cost. (B). Carbon Cap vs Production Quantity. (C) Carbon Cap vs Service Level. (D) Carbon Cap vs Environmental Cost. (E) Carbon Cap vs Social Cost.

According to Kyoto protocol, carbon footprint must decrease in the next years for preserving the ecology and the environment. Recently, US Government announced that the total carbon emissions in the US will be decreased by 32% by 2030 [34]. Other developed nations are also expected to announce similar reductions in their carbon emissions. Therefore, analysis of the manufacturing systems under different carbon emission reduction goals will provide important insight from the triple bottom line accounting perspective of sustainability. Our results show that changing carbon caps leads to changes in the total cost as shown in Fig 2. Fig 2A shows the change in the total cost is proportional to the decrease in the carbon cap. The main reason behind this reduction is not due to efficiency gain, but the decreasing production amount as shown in Fig 2B. The production amount is directly linked to carbon cap; as long as the carbon emission per product does not change, the production amounts must decrease. When production amount decreases, the service level of company decreases as shown in Fig 2C. This decrease in production quantity leads lower service levels. So, carbon cap dictates the production quantity. Production capacity can be increased under carbon cap by improving the resource consumption rate (such as improving the production system to generate less waste and consume less energy) per unit product.

The environmental cost is directly linked to the carbon footprint of the company and electricity consumption: because the amount of production decreases due to carbon cap, environmental cost decreases due to lower production quantities under reduced carbon cap levels. Fig 2D shows the change in the environmental cost with respect to change in the carbon cap.

The relationship between the social cost and the carbon cap is shown in Fig 2E. The social cost decreases with the decreasing carbon cap. When the production amount decreases due to decreasing carbon cap (Fig 2B), the number of employees is enough to operate the manufacturing facility at maximum capacity under carbon cap and no overtime is needed and worker production cost also decreases due to lower production quantity. However, this reduction in the production quantity is reflected as financial cost due to not meeting the demand or a lower service level as shown in Fig 2C.

### Smoothing Limit

**Fig 3:**
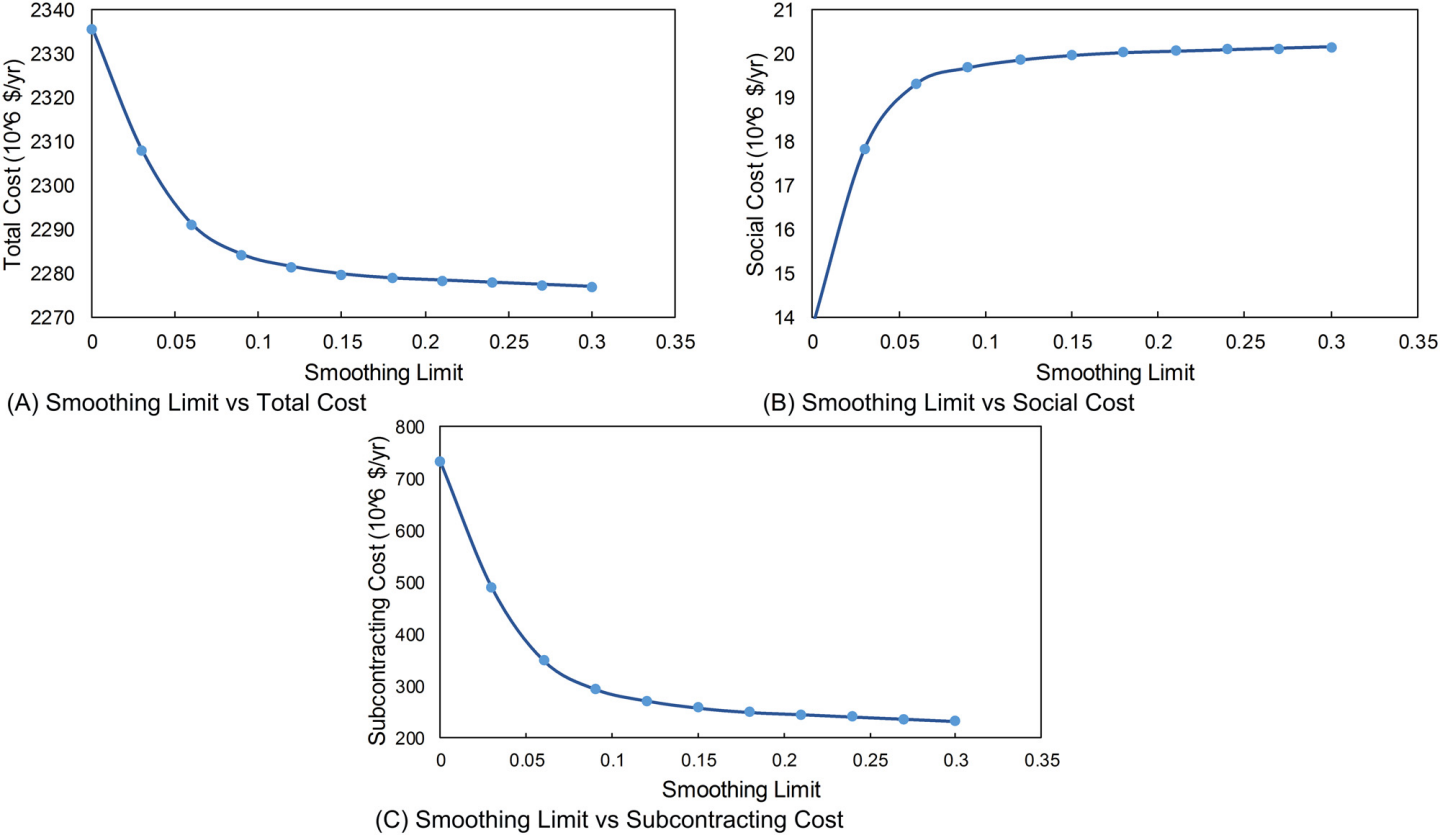
Sensitivity with respect to smoothing limit. (A) Smoothing Limit vs Total Cost. (B) Smoothing Limit vs Social Cost. (C) Smoothing Limit vs Subcontracting Cost.

According ILO, there is an upper limit on the number of people fired and hired for each period. When the smoothing limit changes, the total cost for production also changes. The effect of smoothing limit on the total cost is shown in Fig 3. When the smoothing limit increases up to a certain point, the total cost decreases as seen in Fig 3A. There is a shortage of workers in the manufacturing facility and if the smoothing limit is low, the company cannot hire new workers and needs to subcontract more, leading to an increase in the total cost. When this limit is high, company can hire workers immediately and the social cost increases while smoothing limit increases. Fig 3B shows the change in the social cost with respect to the smoothing limit. The social cost increases when smoothing limit increases because the manufacturer can hire more workers; it starts to manufacture product in its own facilities rather than subcontracting and it costs as social cost to manufacturer. Fig 3C shows the change in the subcontracting cost change with respect to smoothing limit. Lower smoothing limit does not affect the service level due to subcontracting, manufacturer can subcontract its production accounting the service limit targets and demand levels. As shown in Fig 3A, the total cost decreases when smoothing limit increases. In Fig 3C, the subcontracting cost is decreasing because manufacturer can produce its products and producing its own products is cheaper than subcontracting. So, manufacturer does not prefer to subcontract more.

### Overtime Limit

**Fig 4:**
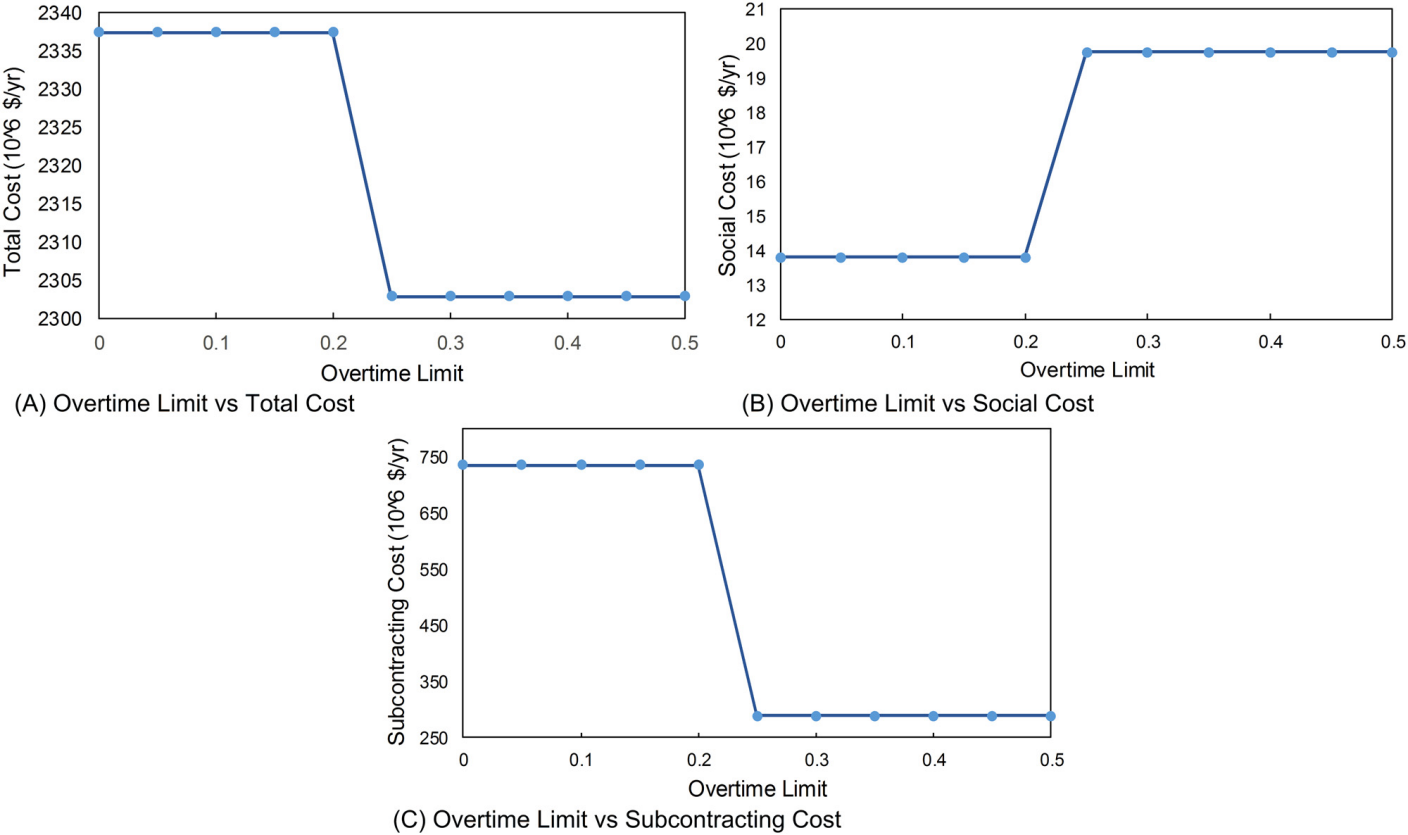
Sensitivity with respect to overtime limit. (A) Overtime Limit vs Total Cost. (B) Overtime Limit vs Social Cost. (C) Overtime Limit vs Subcontracting Cost.

Overtime limit is another factor that affects the social cost and the total cost. If the overtime limit increases, total cost decreases because workers can produce more by overtime as shown in Fig 4. So, smaller number of workers is needed to produce the same amount of product and it decreases the total cost after a breakpoint as shown in Fig 4A. When overtime limit increases, one worker needs to work more when the production capacity without overtime is not enough to satisfy demand in some periods and social cost effect increases. In Fig 4B, the effect of increasing overtime limit is shown. After a breakpoint (which is 17% for this particular case) the total cost decreases although social cost increases. Because subcontracting cost decreases more than social cost increases, the manufacturer wants to produce at its own manufacturing facility instead of subcontracting. A break-point for overtime limit is observed at 17%. This is due to the fact that the manufacturer should hire workers considering the entire planning horizon of one year: if the manufacturer does not employ the required workforce that is used to satisfy the demand with regular and overtime production, company does not want to hire new workers and thus prefers to use subcontracting as seen in Fig 4C. With the overtime limit of 18%, the company can satisfy demand by producing it in its own facilities. The total cost decreases since subcontracting is more expensive than producing, and producing increases the social cost.

We interpret overtime production as an important social factor affecting the life standards of workers more than wages because their life standards decrease due to working more than the standard working hours. But there is no measurement tool and data available to calculate this decline in the standards of living for a worker with overtime.

### The Service Level

**Fig 5:**
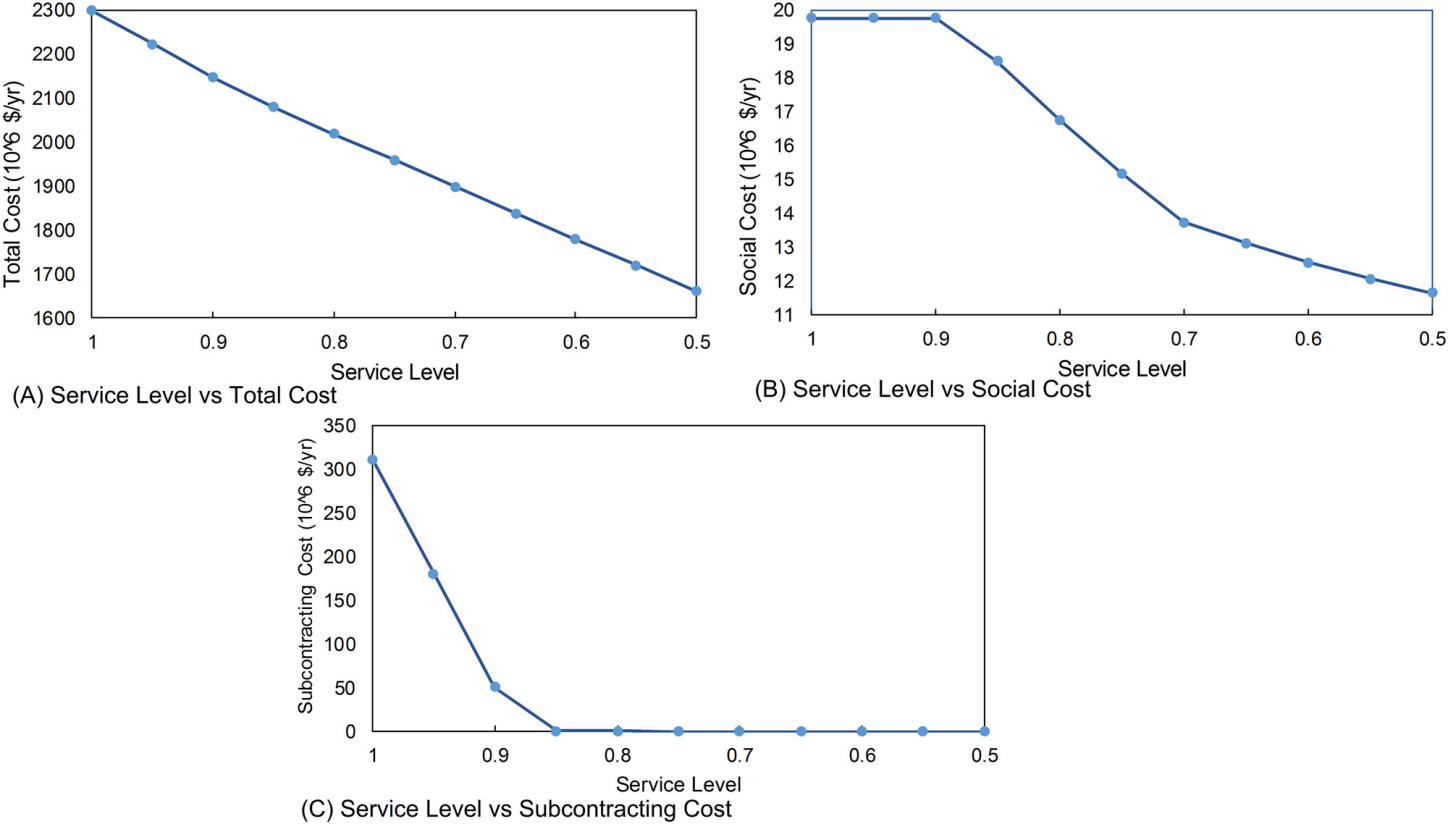
Sensitivity with respect to the service level. (A) Service Level vs Total Cost. (B) Service Level vs Social Cost. (C) Service Level vs Subcontracting Cost.

The service level is directly related to customer demand satisfaction; if manufacturer cannot satisfy the demand, there is a stock-out cost. For the case study that we consider in this paper, the manufacturer can satisfy up to 40% of demand by subcontracting. As shown Fig 5A, when the service level increases, the total cost also increases due to two reasons. One of sources of this increase is the social cost. When the service level increases, the number of workers increases to satisfy the demand. As shown in Fig 5B, the social cost remains stable at high service levels since the necessary production is supplied through subcontracting (see Fig 5C). When the service level decreases, social cost decreases since smaller workforce is needed for production and idle workers are fired; there is no need to employ excess number of workers.

Another factor for the increase in the total cost is subcontracting. After the specific service level, manufacturer cannot satisfy the demand because the workforce is not enough to satisfy the demand; so the manufacturer needs to subcontract. When the service level decreases, manufacturer does not want to subcontract anymore because subcontracting is more expensive than production. In Fig 5C, the subcontracting cost decreases and after the some level of service level manufacturer do not need to subcontract since the manufacturer satisfies the market demand by producing at its own facilities only.

## Discussion and Conclusions

Sustainability and its interpretation in supply chain management is an emerging requirement for decision-makers. The proposed approach is based on the revision of standard mathematical programming (optimization) models of classical aggregate plan with environmental and social factors. It incorporates economic, environmental and social dimensions of sustainability simultaneously: Social and environmental factors are added as the second and third objectives in addition to the economic objective function.

In the aggregate planning model, the decision-maker deduces an optimal production plan that is comprised of the production, inventory, capacity and workforce levels for a finite and medium-term planning horizon with the objective of minimizing the total cost. Standard formulation is revised with additional environmental and social criteria instead of pure-economical comprehension. The numerical results on a case study from real life show that optimal plan changes when such considerations are present. Although it is possible to gain environmental and social benefits, these gains result in an increase in the total economic cost. Furthermore, the analysis also shows that certain legislations and incentives should be placed by regulatory agencies or market mechanisms in order to enforce the adoption of the proposed approach in decision-making.

It is possible to include further extensions to the aggregate planning model for sustainable supply chain model presented in this paper. Here, we consider single commodity model without backorders. The current model can be extended to incorporate multiple commodities and backordering decisions for single or multiple commodities would be interesting extensions of this work.

The contemporary supply chains of the 21^st^ century is required to consider sustainability due to public and legislative pressures. Observing and respecting the public perspective and operating within the legislative restrictions and market requirements is a requirement for public and private organizations. First, the organizations need to identify and measure environmental and social factors that affect the sustainability of their operations. Meanwhile, regulatory agencies need to enforce regulations and policies that consider the triple bottom line accounting of sustainability. The analysis conducted for refrigeration industry in this paper indicates that carbon taxes and caps could be effective in reducing the environmental impact. The social factors are mainly affected by the number of hours worked in a given period such as the number of hours worked in a week. Regulations and restrictions on the number of hours worked and overtime limits are important in achieving sustainability from the social perspective.

When the environmental and social factors are included in the aggregate planning model, the cost structure changes and the optimal policies differ considering three objectives (economic, environmental, and social) rather than conventional economic objective. As shown in Fig 1, it is possible to quantify the changing cost structure when the environmental and social factors are included. The model based approach to aggregate planning of sustainable supply chains presented in this paper gives significant insights into the production planning problem and can be applied to all manufacturing supply chains after collecting the necessary data regarding environmental and social factors.

In this paper, the standard aggregate planning model is modified according to the triple bottom line principles of sustainability. The results show social and environmental factors must be considered as a part of aggregate planning model. When these two factors are considered as a part of aggregate planning model, the total cost of the system increases. In addition, the case study prepared from the real life provides useful insight when the sustainability considerations are included in the decision-making process. This paper aims to show how to create more sustainable production system for electronic industry by taking example of refrigerator manufacturer from Turkey. The decision-makers in the manufacturing and electronic industries can use the modified model to analyze aggregate plans for their operations from sustainability perspective. The manufacturers can implement this model in their production planning system by incorporating environmental and social factors outlined in this paper. Environmental factors can be included considering the energy consumption and greenhouse gas emission per unit product manufactured. The social factors, however, are more challenging to incorporate into the model since they cannot be defined and measured directly. For this purpose, it is necessary to conduct statistical analysis of social factors such as employee job security and morale-motivation, employee health and work-family balance and customer satisfaction on the supply chain. The social cost consists of all worker related costs in the production system. Manufacturers can measure the effect of social factors, by analyzing all worker related costs in their production system.

## Supporting Information

S1 Model The optimization model and the implementation of the *ϵ*–constraint method in the GAMS optimization platform. (GMS)

S1 Table Notation Used in the Model Formulation. (DOCX)

S2 Table Experimental Data for the Case Study. (DOCX)
