# Supplementary material for: Sustainability in Supply Chain Management: Aggregate Planning from Sustainability Perspective
Source: PLoS One. 2016 Jan 25;11(1):e0147502. doi: 10.1371/journal.pone.0147502 (PMC4725800; doi:10.1371/journal.pone.0147502)
Supplement: S1 Table — (DOCX) [file pone.0147502.s002.docx]

**S1 Table. Notation Used in the Model Formulation**

| *Sets* | *Description* |
| --- | --- |
| *T* | Planning horizon (periods) |
| *Variables* | *Description* |
| *W_t_* | Number of workers in period *t* (workers) |
| *H_t_* | Number of workers hired in period *t* (workers) |
| *F_t_* | Number of workers fired in period *t* (workers) |
| *P_t_* | Number of items produced in period *t* (items) |
| *O_t_* | Number of items produced on overtime in period *t* (items) |
| *I_t_* | Inventory on hand in period *t* (items) |
| *SU_t_* | Number of items subcontracted in period *t* (items) |
| *S_t_* | Number of items stock-out in period *t* (items) |
| *Parameters* | *Description* |
| *D_t_* | Demand forecast at each period *t* (items) |
| *H* | Regular time working hours per day (hours/day) |
| *n_t_* | Number of working/production days in period *t* (days) |
| *K* | Number of items produced in one day by one worker (items/day) |
| *W_init_* | Initial workforce level (workers) |
| *I_init_* | Initial inventory level (items) |
| *c_L_* | Cost of labor per hour (money-units/worker) |
| *c_H_* | Cost of hiring a worker (money-units/worker) |
| *c_F_* | Cost of firing a worker (money-units/worker) |
| *c_M_* | Cost of material used in producing one item (money-units/item) |
| *c_O_* | Incremental cost of producing one item (money-units/item) |
| *c_I_* | Cost of holding one item of inventory for one period (money-units/item/period) |
| *c_SU_* | Cost of subcontracting one item (money-units/item) |
| *c_S_* | Cost of stock-out one item (money-units/item) |
| *c_I_^’^* | Amount of GHG emissions per item hold in the inventory (emission-units/item) |
| c*_P_^’^* | Amount of GHG emissions per item produced (emission-units/item) |
| *c_SU_^’^* | Amount of GHG emissions per item subcontracted (emission-units/item) |
| *c_I_^’’^* | Amount of electricity used per item hold in the inventory (electricity-units/item) |
| c*_P_^’’^* | Amount of electricity used per item produced (electricity -units/item) |
| *c_SU_^’’^* | Amount of electricity used per item subcontracted (electricity -units/item) |
| *c_o_^’’^* | Amount of electricity used per item produced in overtime (electricity -units/item) |
| $ɛ$*_C_* | Cap on GHG emissions during the entire planning horizon (emission-units) |
| $ɛ$*_E_* | Cap on electricity consumption during the entire planning horizon (electricity-units) |
| $S$*_lim_* | Smoothing limit for the entire planning horizon (workers) |
| $L$*_lim_* | Layoff limit at each period (workers) |
| $O$*_lim_* | Overtime limit at each period (hours) |
| $\alpha$ | Desired customer service level as a percentage of demand (percentage) |
| *c_c_* | Carbon Tax per Ton |
| *c_c_* | Electricity Cost per Kw/h |
