# Supplementary material for: Sustainability in Supply Chain Management: Aggregate Planning from Sustainability Perspective
Source: PLoS One. 2016 Jan 25;11(1):e0147502. doi: 10.1371/journal.pone.0147502 (PMC4725800; doi:10.1371/journal.pone.0147502)
Supplement: S2 Table — (DOCX) [file pone.0147502.s003.docx]

**S2 Table. Experimental Data for the Case Study**

| *Parameter* | *Base Value* |
| --- | --- |
| *T* | 12 months |
| *t* | a month (*t*$\in${1,…,12}) |
| *D_t_* | [208000, 222000, 268000, 265000, 291500, 250000, 274000, 220000, 278000, 211500, 2342000, 157000] |
| *H* | 7.5 |
| *n_t_* | [21, 20, 23, 21, 22, 22, 21, 20, 23, 21, 22, 22] |
| *K* | 0.66 |
| *W_init_* | 750 |
| *I_init_* | 25000 |
| *c_L_* | 9.52 |
| *c_H_* | 6000 |
| *c_F_* | 8000 |
| *c_M_* | 750 |
| *c_O_* | 1125 |
| *c_I_* | 5 |
| *c_SU_* | 900 |
| *c_S_* | 375 |
| *c_I_^’^* | 0.005 |
| c*_P_^’^* | 0.04 |
| *c_SU_^’^* | 0.04 |
| *c_I_^’’^* | 7 |
| c*_P_^’’^* | 150 |
| *c_SU_^’’^* | 0 |
| *c_o_^’’^* | 150 |
| $ɛ$*_C_* | 150000 |
| $S$*_lim_* | 0.35 |
| $L$*_lim_* | 0.1 |
| $O$*_lim_* | 0.15 |
| $\alpha$ | 0.99 |
| *c_c_* | 100 |
| *c_e_* | 0.2 |
